# Diet quality and cognitive function in mid-aged and older men and women

**DOI:** 10.1186/s12877-019-1326-5

**Published:** 2019-12-21

**Authors:** Catherine M. Milte, Kylie Ball, David Crawford, Sarah A. McNaughton

**Affiliations:** Deakin University, Geelong, Australia, Institute for Physical Activity and Nutrition (IPAN), School of Exercise and Nutrition Sciences, 221 Burwood Highway, Burwood, Vic 3125 Australia

**Keywords:** Diet, Aging, Cognitive function, Diet quality, Dietary guidelines

## Abstract

**Background:**

To date much research into nutrition and cognitive function has been at the nutrient or food level, with inconsistent results. There is increasing interest in the dietary pattern approach to assess whole diet quality and its association with cognitive function. This study investigated if diet quality is associated with cognitive function in men and women aged 55 years and over.

**Methods:**

Adults aged 55–65 years in the Wellbeing, Eating and Exercise for a Long Life (WELL) study in Victoria, Australia (*n* = 617) completed a postal survey including a 111-item food frequency questionnaire in 2010 and 2014. Diet quality was assessed via the revised dietary guideline index (DGI-2013) and also by its individual components which assessed key food groups and dietary behaviours from the Australian Dietary Guidelines. The Telephone Interview of Cognitive Status (TICS-m) measured cognitive function in 2014. Associations between past (2010) and recent (2014) diet quality and its components, and cognitive function were assessed by linear regression adjusted for covariates.

**Results:**

After adjustment for age, sex, education, urban/rural status and physical activity there were no associations between diet quality in 2010 and cognitive function in 2014. However participants who reported higher dietary variety (B = 0.28, 95% CI 0.03, 0.52) and women who reported “sometimes” adding salt to food after cooking (B = 0.98, 95% CI 0.25, 1.71) in 2010 displayed better cognitive function in 2014. In 2014, usual consumption of higher fibre bread choices in the total sample (B = 1.32, 95% CI 0.42, 2.23), and higher diet quality (B = 0.03, 95% CI 0.00, 0.07) and greater fluid consumption (B = 0.14, 95% CI 0.01, 0.27) in men were all associated with better cognitive function. In addition, men who reported “usually” adding salt to their food during cooking displayed poorer cognitive function (B = -1.37, 95% CI -2.39, − 0.35). There were no other associations between dietary intake and cognitive function observed in the adjusted models.

**Conclusion:**

An association between dietary variety and some limited dietary behaviours and cognitive function was observed, with variation by gender. Future research should consider trajectories of dietary change over longer time periods as determinants of health and function in older age.

## Background

The brain undergoes changes with age, including a steady decrease in brain size [1]. This is accompanied by age-related cognitive decline, a process characterised by a gradual decline from mid adulthood onwards in cognitive functions including processing speed, reasoning, memory and executive function. Decline in cognitive function varies in severity and trajectory between individuals. By middle age, and a large variation in cognitive function can be observed [2]. An estimated 25% of the variation is due to genes [3], leaving a large proportion potentially due to modifiable risk factors, including diet. Nutrients, including long-chain omega-3 fatty acids, antioxidants and B vitamins play important roles in the structure and function of the brain [4]. However, to date, results of randomised controlled trials investigating individual nutrients or food groups on cognitive function have been mixed [5].

To date much of the research into nutrition and cognitive function has been at the single nutrient or food level. In contrast the dietary pattern approach has received less attention in the field [5]. There are two overarching categories of dietary patterns approaches: data driven approaches which use multivariate statistical techniques such as factor or cluster analysis; and diet quality indices or dietary scoring methods which are based on a priori guidelines. Diet quality indices can assess adherence to dietary guidelines and/or current evidence about the best diet for good health [6], or a specific traditional or cultural diet such as the Mediterranean diet [7]. Diet quality has been associated with cardio-metabolic risk factors [8], physical and mental health [9] and cogntive function [10].

To date, most studies of dietary patterns and cognitive function have investigated diet at only one point in time [10, 11], and few studies have investigated dietary intake from multiple perspectives including diet quality indices, key food groups and dietary behaviours considered to be part of a healthy or unhealthy dietary intake within one cohort. Considering the inconsistent findings from research to date, assessing diet quality, foods and dietary behaviours at multiple time points in one sample may provide insights into the role of diet in supporting cognitive function in older age. The aim of this study was to investigate if diet quality over 4 years was associated with cognitive function in community dwelling men and women aged 55 years and over. Associations between key food groups, dietary behaviours and cognitive function were also investigated.

## Methods

### Design

This study is based on data from the Wellbeing, Eating and Exercise for a Long Life (WELL) study, a prospective, population-based longitudinal cohort study. WELL was a voluntary survey designed to investigate of nutrition and physical activity behaviours, obesity and quality of life, and the influences on these among mid-aged and older adults at the peri retirement stage [12]. The Australian Electoral Roll was used to select potential community-dwelling participants aged between 55 and 65 years living in urban or rural Victoria. Selected potential participants were stratified by socioeconomic position using the Socioeconomic Index for Areas score (SEIFA) [13]. Of the 11,256 surveys sent to potential participants in 2010, 475 were returned (95 from individuals outside the age bracket and 380 were undeliverable). There were 4082 participants who completed surveys and provided informed consent by return of the survey at baseline (response rate 38%). A follow-up survey was sent in 2014 to participants who had agreed to be contacted again in the previous wave (*n* = 3123) and 2542 completed surveys were returned (response rate 81%). The surveys were sent at the same time of year in 2010 and 2014 to negate any potential seasonal effects. The Deakin University Human Research Ethics Committee (2009–105) provided ethical approval for the study. Full details of the study have been described elsewhere [12].

In 2014, cognitive function data was collected in a subgroup of participants during a brief telephone interview. Those WELL study participants who had completed the 2014 follow-up written survey and were living in urban or urban fringe suburbs were invited to take part. A sample of *n* = 1117 were sent an invitation pack to participate in the telephone interview, with 808 providing informed written consent to take part (72% response rate). A total of 745 telephone interviews were completed.

### Cognitive function

Cognitive function was assessed during the telephone interview using the Telephone Interview for Cognitive Status Modified (TICS-m) [14]. TICS-m is a brief, 13-item test of global cognitive function with scores ranging from 0 to 50 with lower scores reflecting greater cognitive impairment. A score of ≥32 indicates normal cognitive function, with scores between 31 and 28 and ≤ 27 indicating possible mild cognitive impairment and dementia respectively [15]. Items cover a range of cognitive tasks including orientation, repetition, naming, and calculations. The TICS-m also includes a 10-item non-semantically related word list which participants are asked to recall both immediately and after a delay of about five minutes filled with distractor questions. The current study used a version previously adapted for the Australian population [16, 17].

### Dietary intake

Self-reported dietary intake was assessed using a food frequency questionnaire (FFQ) of 111 food and beverage items over the last six months [18, 19]. The FFQ was adapted from other national studies [20, 21], has been used to assess diet quality and demonstrated as a good predictor of health outcomes previously [6]. Intake of items were converted into daily equivalent frequencies for scoring of diet quality [22]. To assess general food habits and dietary behaviours the survey also included seven additional validated short questions including salt use (during and after cooking), type of milk and bread consumed, trimming the fat from meat and daily fruit and vegetable consumption [23].

### Diet quality

Diet quality was assessed using the dietary guideline index (DGI-2013) [24]. The DGI-2013 assesses adherence to the 2013 Australian Dietary Guidelines [25], updated from a previous version of the DGI [6]. The DGI-13 consists of 13 components scored from 0 (not meeting recommendation) to 10 (fully meeting recommendation), using age- and sex-specific cut-offs from the Australian Dietary Guidelines [25]. The DGI-13 assesses consumption of five core food groups (vegetables, fruits, grains, meat and alternatives, and dairy), fluids and discretionary foods. The index also includes items assessing consumption of dietary variety, lean protein, reduced−/low-fat dairy, whole-grain cereals and unsaturated fats and oils. The 13 items are summed so that the total diet score has a possible range of 0 to 130, with higher scores reflecting greater diet quality. The DGI-2013 was shown to be related to sociodemographic factors, health behaviours, self-assessed health, and markers of cardio-metabolic health in previously [9, 24, 26]. In addition to the overall diet score, individual components of the DGI-2013 which assessed key food groups and dietary behaviours were also included in the analysis. See Additional file 1: Table S1 for details of the DGI-2013 and individual components included in the analysis.

### Covariates

Participant characteristics including date of birth for calculation of age, marital status, retirement status, smoking status, country of birth and highest education level achieved were collected during the survey. Body mass index (BMI; kg/m^2^) was calculated from self-reported height and weight and standard cut points applied to determine overweight and obesity status [27]. The self-administered International Physical Activity Questionnaire (IPAQ-L) assessed total physical activity during the preceeding 7 days. A previous 12-country, 14-site study determined the validity and reliability of IPAQ-L [28]. Items relating to assesses duration, frequency and intensity of leisure, work, commuting and household/yard were collected and responses converted into total metabolic equivalent of task (MET) hours per week. Moderate physical activity was set at 3 MET and vigorous physical activity was set at 6 MET. Self-reported past history of cardiovascular disease (stroke, diabetes, heart disease and hypertension) were also collected. All potential covariates were collected at both baseline and follow up, with the exception of date of birth, country of birth and education status, which were only collected at baseline.

### Statistical analysis

Participants missing > 10% responses on the FFQ, or one or more responses to the dietary habits questions, the TICS-m score or covariates were excluded from analysis. Characteristics of study participants were described using summary statistics. Difference in DGI score at 2010 and 2014 was assessed using paired t-test. Characteristics of included and excluded participants were compared using independent t-test and chi square and presented in Additional file 1: Table S2. For the main analysis, associations between diet quality and individual DGI-2013 components in 2010 (past dietary intake) and 2014 (recent dietary intake) and TICS-m in 2014 were assessed via multivariate linear regression with TICS-m as a continuous variable. Potential covariates adjusted for in regression models were determined by previous literature [6, 29], and those associated with the outcome and exposure were included in the models. However, BMI, depression and history of cardiovascular disease were not included in the adjusted regression model as they were deemed to be on the potential casual pathway between poor diet and cognitive function. Adding a confounder which is on the causal pathway can result in over adjustment bias, where the true relationship between exposure and outcome cannot be estimated [30]. Multivariable linear regression models were adjusted for age, sex, education, urban/rural location and total physical activity. The main analysis was conducted in the total sample and men and women separately as a previous study of diet quality and cognitive function have reported differences by sex [31]. A sensitivity analysis was also conducted where the main regression analysis was repeated excluding participants who had reported cardiovascular disease or stroke (see Additional file 1: Table S3 and S4). StataSE version 13.1 (StataCorp, TX, USA) was used for all statistical analyses. *P* < 0.05 was considered significant.

## Results

There was complete data available for analysis on 617 participants (Table 1). Most participants were born in Australia (77%), married or partnered (77% in 2010, 75% in 2014) and had a university degree (45%). Although only 35% of participants were retired at baseline, by 2014, 55% of the sample were retired, reflective of the selected age group and life stage. There were 354 (57%) participants in 2010 and 361 (58%) participants in 2014 with a BMI classified as overweight or obese. The mean (SD) DGI-2013 score was 87.5 (13.9) at baseline, indicating moderate levels of diet quality in the sample. Diet quality remained stable over the four years, with similar DGI-2013 scores reported in 2010 and 2014. The mean (SD) TICS-m score was 37.0 (4.06) out of a possible 50 points, indicating normal cognitive function in the sample overall (Table 1). Scores ranged from 24 to 48 points in the sample and scores were similar for men and women. Only 61 participants (10%) had scores below 32 points, indicating possible mild cognitive impairment or dementia.

**Table 1:** Characteristics of 617 Men and Women from the WELL Study, Victoria, Australia, 2010–2014

	Total *n* = 617	Men *n* = 302	Women *n* = 315
	Mean (SD)	Mean (SD)	Mean (SD)
Age T1 (years)	60.2 (3.14)	60.2 (3.11)	60.2 (3.17)
BMI T1 (kg/m^2^)	26.6 (4.71)	27.2 (4.31)	26.1 (5.00)
BMI T3 (kg/m^2^)	26.5 (4.76)	27.1 (4.31)	26.0 (5.09)
Total physical activity T1 (MET hours/week)	93.1 (78.9)	97.2 (82.8)	89.1 (74.8)
Total physical activity T3 (MET hours/week)	93.8 (82.4)	97.2 (84.5)	90.6 (80.4)
Dietary Guideline Index T1	87.5 (13.9)	84.4 (13.9)	90.5 (13.2)
Dietary Guideline Index T3	88.2 (13.3)	85.2 (13.5)	91.1 (12.4)
Change in Dietary Guideline Index T3-T1	0.66 (11.0)	0.77 (10.3)	0.55 (11.6)
Geriatric depression scale T3	1.72 (2.30)	1.71 (2.25)	1.73 (2.34)
TICS-M T3	37.0 (4.06) Min – 24 Max - 48	36.3 (3.86) Min – 24 Max - 48	37.6 (4.15) Min – 24 Max - 48
Region T1	N (%)		
*Urban*	598 (96.9)	293 (97.0)	305 (96.8)
*Rural*	19 (3)	9 (2.98)	10 (3.17)
Country of birth			
*Australia*	472 (76.5)	221 (73.2)	251 (79.7)
*UK*	49 (7.94)	31 (10.26)	18 (5.71)
*Other*	96 (15.5)	50 (16.6)	46 (14.6)
Relationship status T1			
*Married/defacto*	477 (77.4)	258 (85.4)	219 (69.6)
*Separated/divorced*	82 (13.3)	25 (8.28)	57 (18.2)
*Widowed*	28 (4.55)	3 (0.99)	25 (7.96)
*Never married*	29 (4.71)	16 (5.30)	13 (4.14)
Retired T1			
*Yes*	215 (35.3)	87 (29.1)	128 (41.2)
*No*	395 (64.8)	212 (70.9)	183 (58.8)
Education			
*Up to 10 years*	136 (22.0)	51 (16.9)	85 (27.0)
*12 years/trade/certificate*	204 (33.1)	116 (38.4)	88 (27.9)
*University degree*	277 (44.9)	135 (44.7)	142 (45.1)
Smoking status T1			
*Never smoked*	346 (56.1)	154 (51.0)	192 (60.9)
*Former smoker*	207 (33.6)	120 (39.7)	87 (27.6)
*Daily smoker*	64 (10.4)	28 (9.27)	36 (11.4)
History of stroke, n (%)	18 (2.9)	11 (3.6)	7 (2.2)
History of diabetes, n (%)	52 (8.4)	37 (12.3)	15 (4.8)
History of heart disease, n (%)	112 (18.2)	73 (24.2)	39 (12.4)
History of hypertension, n (%)	276 (44.7)	139 (46.0)	137 (43.5)

### Associations between past (2010) diet intake and cognitive function

Table 2 shows the association between past diet quality and components in 2010 and TICS-m in 2014 assessed by multiple linear regression. In the total sample, higher diet quality assessed by the DGI-2013 (indicating greater adherence to the Australian Dietary Guidelines) was associated with better cognitive function in the crude model (B = 0.03, 95% CIs 0.00, 0.05). However this association did not remain significant after adjustment for age, sex, education, urban/rural status and total physical activity. Higher dietary variety was also associated with better cognitive function in the total sample, and remained significant after adjustment for confounders (B = 0.28, 95% CIs 0.03, 0.52). An association between wholegrain bread and cognitive function was observed but this did not remain significant in the adjusted model. There were no other associations between past dietary intake and cognitive function in the total sample.

**Table 2:** Multivariate regression for DGI-2013 and components (2010) vs. TICS-m 2014 in the WELL study

	Total (*n* = 617)	Men (*n* = 302)	Women (*n* = 315)
	B (95% CI)	B (95% CI)	B (95% CI)
DGI-2013			
Crude	0.03 (0.00, 0.05)*	0.02 (−0.01, 0.05)	0.01 (− 0.03, 0.04)
Adjusted	0.00 (− 0.01, 0.03)	0.02 (− 0.01, 0.05)	0.00 (− 0.03, 0.04)
Dietary variety			
Crude	0.41 (0.14, 0.67)**	0.37 (− 0.04, 0.77)	0.30 (− 0.04, 0.65)
Adjusted	0.28 (0.03, 0.52)*	0.31 (− 0.04, 0.65)	0.26 (− 0.06, 0.59)
Vegetable serves			
Crude	0.27 (−0.01, 0.56)	0.10 (−0.17, 0.38)	0.20 (− 0.27, 0.68)
Adjusted	0.20 (−0.08, 0.47)	0.20 (− 0.08, 0.48)	0.27 (− 0.20, 0.74)
Fruit serves			
Crude	0.05 (−0.15, 0.25)	0.05 (−0.29, 0.39)	− 0.16 (− 0.52, 0.20)
Adjusted	−0.06 (− 0.28, 0.17)	0.07 (− 0.24, 0.38)	− 0.16 (− 0.53, 0.21)
Grain foods serves			
Crude	−0.07 (− 0.40, 0.26)	0.07 (− 0.22, 0.36)	−0.08 (− 0.55, 0.40)
Adjusted	− 0.05 (− 0.34, 0.25)	0.03 (− 0.26, 0.32)	− 0.17 (− 0.59, 0.25)
Wholegrain bread			
Crude			
*“I don’t eat bread/white bread”*	ref	ref	ref
*“high fibre white/multigrain/other”*	1.31 (0.37, 2.26)**	0.60 (− 0.44, 1.64)	1.92 (0.35, 3.49)*
*“Wholemeal/rye”*	0.28 (−0.77, 1.33)	− 0.76 (−2.06, 0.54)	1.52 (0.09, 2.95)*
Adjusted			*n* = 311
*“I don’t eat bread/white bread”*	ref	ref	ref^a^
*“high fibre white/multigrain/other”*	0.87 (−0.05, 1.79)	0.46 (− 0.54, 1.47)	1.29 (− 0.08, 2.66)
*“Wholemeal/rye”*	− 0.03 (− 1.06, 1.00)	− 0.79 (− 2.11, 0.53)	0.95 (− 0.44, 2.34)
Meat and alternatives serves			
Crude	0.17 (− 0.15, 0.49)	0.23 (− 0.14, 0.60)	0.05 (− 0.29, 0.39)
Adjusted	0.03 (− 0.27, 0.34)	0.17 (− 0.17, 0.52)	− 0.03 (− 0.38, 0.31)
Dairy serves			
Crude	0.18 (− 0.06, 0.43)	0.13 (− 0.26, 0.52)	0.18 (− 0.23, 0.59)
Adjusted	0.18 (− 0.06, 0.43)	0.11 (− 0.26, 0.47)	0.18 (− 0.21, 0.57)
Fluid serves			
Crude	0.08 (− 0.00, 0.16)	0.11 (− 0.02, 0.24)	− 0.01 (− 0.13, 0.12)
Adjusted	0.01 (− 0.06, 0.09)	0.06 (− 0.09, 0.20)	− 0.03 (− 0.15, 0.09)
Discretionary foods serves			
Crude	−0.04 (− 0.14, 0.07)	0.08 (− 0.04, 0.20)	− 0.03 (− 0.19, 0.14)
Adjusted	0.04 (− 0.06, 0.13)	0.07 (− 0.05, 0.19)	−0.01 (− 0.17, 0.14)
Trimming fat from meat			
Crude			
*“Never/rarely”*	ref	ref	ref
*“Sometimes”*	0.43 (− 1.29, 2.15)	0.37 (− 1.71, 2.44)	0.18 (−3.31, 3.67)
*“Usually/always/I don’t eat meat”*	1.11 (−0.50, 2.72)	0.64 (− 1.26, 2.55)	0.97 (− 1.93, 3.86)
Adjusted			
*“Never/rarely”*	ref	ref	ref
*“Sometimes”*	−0.08 (−1.59, 1.42)	− 0.02 (−2.08, 2.04)	− 0.34 (−3.76, 3.07)
*“Usually/always/I don’t eat meat”*	0.37 (− 1.06, 1.81)	0.36 (− 1.53, 2.24)	0.34 (− 2.34, 3.02)
Type of milk drunk			
Crude			
*“Whole”*	ref	ref	ref
*“Don’t know/soy”*	0.20 (−1.34, 1.74)	−0.85 (−3.04, 1.34)	0.16 (−2.08, 2.41)
*“I don’t drink milk/low fat/skim”*	0.27 (−0.50, 1.04)	−0.16 (−1.06, 0.75)	0.33 (− 1.13, 1.80)
Adjusted			
*“Whole”*	ref	ref	ref
*“Don’t know/soy”*	−0.67 (−1.89, 0.54)	−1.00 (−3.34, 1.33)	−0.52 (−2.39, 1.35)
*“I don’t drink milk/low fat/skim”*	−0.20 (− 0.99, 0.59)	−0.14 (− 1.01, 0.72)	−0.27 (− 1.80, 1.26)
Unsaturated fat oils and spreads serves			
Crude	0.31 (−0.03, 0.65)	0.34 (−0.01, 0.70)	0.26 (− 0.14, 0.65)
Adjusted	0.19 (−0.15, 0.53)	0.26 (− 0.09, 0.60)	0.14 (− 0.28, 0.55)
Salt added after cooking			
Crude			
*“Never”*	ref	ref	ref
*“Sometimes”*	0.50 (−0.19, 1.18)	− 0.22 (−1.18, 0.73)	1.14 (0.26, 2.02)*
*“Usually”*	−0.05 (−1.02, 0.92)	− 0.64 (−2.00, 0.73)	0.75 (− 0.29, 1.79)
Adjusted	*n* = 606		*n* = 310
*“Never”*	ref^a^	ref	ref^a^
*“Sometimes”*	0.34 (−0.23, 0.91)	0.05 (− 0.99, 1.08)	0.98 (0.25, 1.71)*
*“Usually”*	0.23 (−0.72, 1.19)	−0.18 (−1.58, 1.23)	0.63 (− 0.23, 1.49)
Salt added during cooking			
Crude			
*“Never”*	ref	ref	ref
*“Sometimes”*	0.08 (− 0.61, 0.76)	0.44 (−0.60, 1.48)	− 0.11 (−1.22, 0.99)
*“Usually”*	−1.06 (−2.21, 0.09)	− 1.35 (− 2.82, 0.12)	−0.64 (− 2.23, 0.94)
*“Don’t know”*	−2.23 (−4.78, 0.32)	−1.52 (−4.04, 0.99)	n/a
Adjusted			
*“Never”*	ref	ref	ref
*“Sometimes”*	0.12 (−0.54, 0.78)	0.26 (− 0.76, 1.28)	0.01 (−1.11, 1.14)
*“Usually”*	−0.91 (−1.96, 0.14)	−1.26 (−2.72, 0.21)	−0.49 (− 1.96, 0.97)
*“Don’t know”*	−1.60 (−4.31, 1.12)	−1.51 (− 4.45, 1.44)	n/a
High sugar food serves			
Crude	−0.29 (− 0.52, − 0.06)	−0.17 (− 0.41, 0.06)	−0.25 (− 0.91, 0.40)
Adjusted	− 0.16 (− 0.35, 0.04)	−0.15 (− 0.36, 0.05)	−0.17 (− 0.73, 0.39)
Alcohol serves			
Crude	0.10 (−0.13, 0.34)	0.22 (0.01, 0.44)*	0.36 (−0.23, 0.94)
Adjusted	0.23 (− 0.01, 0.46)	0.22 (−0.04, 0.47)	0.27 (− 0.24, 0.78)

Adjusted for age (sex – total only) + education + urban/rural + clustering by postcode + total physical activity (T1)

*DGI-2013* Dietary Guideline Index, *TICS-m* Telephone Interview of Cognitive Status

^a^Results presented with outliers removed, *P < 0.05, **P < 0.01

In sex-stratified analyses, there were no significant associations between past diet quality or its components in 2010 and cognitive function in 2014 in men in the adjusted models. In women, participants who reported “sometimes” adding salt to their food after cooking reported better cognitive function than participants who reported “never” in the adjusted models (B = 0.98, 95% CI 0.25, 1.71). There were no other significant associations reported between past dietary intake and current cognitive function after adjustment for confounders in women.

### Associations between recent (2014) dietary intake and cognitive function

Table 3 shows the associations between recent dietary intake in 2014 and TICS-m. In the crude model for the total sample, there were significant associations observed between diet quality assessed by the DGI-2013, vegetable serves, added salt during cooking and cognitive function, however none of these remained significant after adjustment for age, sex, education, urban/rural status and total physical activity. Usual consumption of higher fibre or multigrain bread types was associated with better cognitive function compared to white bread and remained significant after adjustment of confounders (B = 1.32, 95% CI 0.42, 2.23). There were no other associations between dietary intake and cognitive function in the total sample.

**Table 3:** Multivariate regression for DGI-2013 and components (2014) vs. TICS-m 2014 in the WELL study

	Total (*n* = 617)	Men (*n* = 302)	Women (*n* = 315)
	B (95% CI)	B (95% CI)	B (95% CI)
DGI-2013			
Crude	0.03 (0.01, 0.05)*	0.04 (0.01, 0.08)**	− 0.01 (− 0.05, 0.03)
Adjusted	0.01 (− 0.01, 0.04)	0.03 (0.00, 0.07)*	− 0.01 (− 0.04, 0.03)
Dietary variety			
Crude	0.06 (− 0.20, 0.33)	0.09 (− 0.24, 0.42)	− 0.09 (− 0.44, 0.27)
Adjusted	0.02 (− 0.21, 0.24)	0.09 (− 0.17, 0.35)	− 0.05 (− 0.36, 0.26)
Vegetable serves			
Crude	0.33 (0.09, 0.57)*	0.23 (− 0.06, 0.52)	0.22 (− 0.15, 0.58)
Adjusted	0.21 (− 0.01, 0.43)	0.23 (− 0.04, 0.51)	0.21 (− 0.13, 0.55)
Fruit serves			
Crude	0.07 (− 0.15, 0.30)	0.09 (− 0.20, 0.39)	− 0.16 (− 0.75, 0.42)
Adjusted	− 0.03 (− 0.28, 0.21)	0.11 (− 0.17, 0.40)	− 0.20 (− 0.75, 0.35)
Grain foods serves			
Crude	−0.24 (− 0.52, 0.04)	0.21 (− 0.02, 0.44)	− 0.58 (− 1.00, − 0.16)**
Adjusted	−0.18 (− 0.46, 0.09)	0.12 (− 0.11, 0.34)	*n* = 305^a^ − 0.29 (− 0.74, 0.15)
Wholegrain bread			
Crude			
*“I don’t eat bread/white bread”*	ref	ref	ref
*“high fibre white/multigrain/other”*	1.52 (0.53, 2.51)**	1.28 (0.35, 2.22)**	1.69 (− 0.05, 3.43)
*“Wholemeal/rye”*	1.26 (0.26, 2.27)*	0.47 (− 0.90, 1.83)	2.15 (0.63, 3.66)**
Adjusted			
*“I don’t eat bread/white bread”*	*n* = 605 ref^a^	*n* = 294 ref^a^	ref
*“high fibre white/multigrain/other”*	1.32 (0.42, 2.23)*	1.06 (−0.01, 2.12)	1.35 (− 0.36, 3.06)
*“Wholemeal/rye”*	0.83 (−0.12, 1.79)	0.47 (− 0.75, 1.70)	1.25 (− 0.18, 2.69)
Meat and alternatives serves			
Crude	0.16 (− 0.16, 0.48)	0.11 (−0.26, 0.49)	0.13 (− 0.33, 0.59)
Adjusted	0.11 (− 0.15, 0.38)	0.12 (− 0.21, 0.46)	0.11 (− 0.27, 0.50)
Dairy serves			
Crude	0.11 (− 0.05, 0.27)	0.29 (0.04, 0.53)	− 0.08 (− 0.37, 0.21)
Adjusted	0.12 (− 0.06, 0.30)	0.24 (− 0.01, 0.48)	0.03 (− 0.32, 0.37)
Fluid serves			
Crude	0.09 (−0.02, 0.20)	0.20 (0.09, 0.31)**	−0.06 (− 0.24, 0.12)
Adjusted	0.05 (−0.06, 0.16)	0.14 (0.01, 0.27)*	−0.01 (− 0.20, 0.18)
Discretionary foods serves			
Crude	−0.05 (− 0.20, 0.11)	0.02 (− 0.11, 0.15)	0.07 (− 0.24, 0.38)
Adjusted	0.06 (− 0.10, 0.22)	0.06 (− 0.07, 0.19)	0.07 (− 0.25, 0.38)
Trimming fat from meat			
Crude			
*“Never/rarely”*	ref	ref	ref
*“Sometimes”*	0.48 (−0.87, 1.83)	0.77 (− 0.58, 2.12)	− 0.02 (−2.60, 2.55)
*“Usually/always/I don’t eat meat”*	0.74 (− 0.21, 1.68)	0.25 (− 0.85, 1.36)	0.87 (− 0.31, 2.04)
Adjusted			
*“Never/rarely”*	ref	ref	ref
*“Sometimes”*	0.77 (−0.60, 2.13)	1.18 (− 0.18, 2.53)	− 0.01 (−2.60, 2.58)
*“Usually/always/I don’t eat meat”*	0.38 (− 0.30, 1.06)	0.28 (− 0.63, 1.19)	0.48 (− 0.71, 1.68)
Type of milk drunk			
Crude			
*“Whole”*	ref	ref	ref
*“Don’t know/soy”*	−0.37 (−1.66, 0.92)	− 0.76 (−2.54, 1.02)	−0.94 (− 2.86, 0.98)
*“I don’t drink milk/low fat/skim”*	0.52 (− 0.14, 1.18)	0.50 (− 0.39, 1.38)	0.17 (− 0.89, 1.23)
Adjusted			
*“Whole”*	ref	ref	ref
*“Don’t know/soy”*	−0.90 (−2.20, 0.39)	− 0.91 (− 2.45, 0.63)	−0.99 (− 2.85, 0.87)
*“I don’t drink milk/low fat/skim”*	0.23 (− 0.46, 0.92)	0.48 (− 0.35, 1.31)	−0.03 (− 1.09, 1.03)
Unsaturated fat oils and spreads serves			
Crude	0.21 (−0.16, 0.58)	−0.04 (− 0.54, 0.46)	0.61 (0.19, 1.02)**
Adjusted	0.16 (−0.16, 0.48)	− 0.05 (− 0.47, 0.37)	*n* = 314^a^ 0.41 (− 0.02, 0.84)
Salt added after cooking			
Crude			
*“Never”*	ref	ref	ref
*“Sometimes”*	−0.03 (− 0.70, 0.65)	−0.52 (−1.50, 0.45)	0.44 (− 0.58, 1.47)
*“Usually”*	− 0.17 (− 1.02, 0.68)	−0.73 (− 2.21, 0.74)	0.58 (− 0.38, 1.54)
Adjusted			
*“Never”*	ref	ref	ref
*“Sometimes”*	0.07 (−0.59, 0.73)	−0.27 (−1.29, 0.76)	0.41 (− 0.58, 1.40)
*“Usually”*	0.11 (− 0.79, 1.00)	−0.32 (− 1.83, 1.18)	0.54 (− 0.33, 1.41)
Salt added during cooking			
Crude			
*“Never”*	ref	ref	ref
*“Sometimes”*	−0.49 (−1.29, 0.32)	− 0.88 (− 1.88, 0.13)	−0.11 (− 1.24, 1.02)
*“Usually”*	−1.41 (−2.51, − 0.30)*	−1.95 (−3.12, − 0.78)**	−0.78 (− 2.24, 0.68)
*“Don’t know”*	−1.45 (−4.15, 1.25)	−1.03 (− 3.82, 1.76	n/a
Adjusted	*n* = 612	n = 294	
*“Never”*	ref^a^	ref^a^	ref
*“Sometimes”*	−0.49 (−1.14, 0.16)	−0.65 (− 1.48, 0.18)	−0.13 (− 1.26, 0.99)
*“Usually”*	−1.12 (−2.04, − 0.19)	−1.37 (− 2.39, − 0.35)*	−0.47 (− 1.70, 0.76)
*“Don’t know”*	− 0.93 (− 2.98, 1.13)	−0.91 (− 3.04, 1.21)	n/a
High sugar food serves			
Crude	−0.41 (− 0.83, 0.01)	−0.22 (− 0.60, 0.16)	−0.35 (− 1.55, 0.85)
Adjusted	− 0.18 (− 0.57, 0.21)	−0.07 (− 0.44, 0.30)	−0.42 (− 1.44, 0.60)
Alcohol serves			
Crude	0.07 (−0.15, 0.29)	0.15 (−0.12, 0.43)	0.42 (− 0.12, 0.96)
Adjusted	*n* = 613^a^ 0.14 (−0.117, 0.46)	0.20 (− 0.14, 0.54)	0.55 (− 0.11, 1.21)

Adjusted for age (sex – total only) + education + urban/rural + clustering by postcode + total physical activity (T1)

*DGI-2013* Dietary Guideline Index, *TICS-m* Telephone Interview of Cognitive Status

^a^Results presented with outliers removed, *P < 0.05, **P < 0.01

In men, a higher current DGI-2013 score was associated with a better cognitive function after adjustment for confounders (B = 0.03, 95% CI 0.003, 0.006). A higher consumption of fluids was also associated with better cognitive function in the adjusted model (B = 0.14, 95% CI 0.01, 0.27). After adjustment for confounders, men who reported “usually” adding salt to their food during cooking displayed poorer cognitive function than men who never added salt (B = -1.41, 95% CI -2.51, − 0.30). There were no other significant associations reported between current dietary intake and cognitive function in men, and no associations between current dietary intake and cognitive function in women in the adjusted models.

## Discussion

This study examined associations between diet quality, key food groups and dietary behaviours and cognitive function cross-sectionally and over four years in 617 mid and early older aged men and women. After adjustment for age, sex, education, urban/rural status and total physical activity participants who reported higher dietary variety in the total sample and women who reported “sometimes” adding salt to food after cooking in 2010 displayed better cognitive function in 2014. In 2014, usual consumption of higher fibre bread choices in the total sample, and higher diet quality and greater fluid consumption in men were all associated with better cognitive function. In addition, men who reported “usually” adding salt to their food during cooking displayed poorer cognitive function. There were no other associations between dietary intake and cognitive function observed in the adjusted models. Overall there were limited associations found between dietary intake and cognitive function which may be explained by the low levels of cognitive impairment in this sample of adults from mid and early older age.

Whilst a range of previous studies have investigated associations between diet quality and cognitive function, the majority of these have been in people aged 65 years and over and assessed adherence to a Mediterranean style diet as a form of diet quality [11]. With observable cognitive changes starting to occur in early to mid-adulthood and beta-amyloid protein accumulation observed decades before cognitive impairment or dementia occur [1], it is likely that lifestyle-associated changes in cognitive function have already occurred by age 65. In contrast, there have been fewer studies of diet quality and cognitive function in mid and early older age, with mixed findings. One cross-sectional study of 1269 Puerto Rican men and women residing in Boston found that higher adherence to a Mediterranean Diet and American Dietary Guidelines was associated with higher Mini-Mental State Examination (MMSE) scores, a measure of overall cognitive function [10]. However a larger study of 3083 French adults aged 45 years and over assessed adherence to the Mediterranean diet and cognitive function via a battery of 6 neuropsychological tests over 13 years, and found only limited associations between Mediterranean diet adherence and better cognitive performance on two cognitive subtests of different domains and no associations with global cognitive function overall [32]. The current study has also observed inconsistent results between diet quality and cognitive function, and it is possible that the types of cognitive assessments used to date are ineffective at detecting the fine-grade variation in cognitive function which is observed at this life stage.

The current study not only investigated relationships between diet quality and cognitive function, but also key food groups and dietary behaviours to provide a comprehensive assessment of dietary intake. However there was a lack of consistency in the relationships observed between overall diet quality, the other dietary items and cognitive function, with many individual dietary items not related to cognitive function. A previous study of the Mediterranean Diet and MMSE in men and women from Greece aged 65 years and over also reported associations with the overall score which were largely not reflected in the individual items [31]. Together these findings suggest that the overall diet quality score or whole dietary pattern is greater than the sum of its parts and may be more important to overall health than the individual items alone.

There are several plausible underlying mechanisms which could be driving the associations between components of dietary intake and cognitive function observed in this study. Firstly, higher diet quality could reduce chronic diseases such as cardiovascular disease and diabetes [33], outcomes that have been associated with reduced cognitive impairment [34]. The DGI-2013 is based on recommendations from the Australian Dietary Guidelines which are designed to promote health and reduce chronic disease. The previous DGI was associated with a favourable dietary intake, including higher intakes of fibre, β-carotene, vitamin-C, folate, calcium and iron and lower intakes of energy, total fat and saturated fat [6].

An emerging theory is that consumption of a healthier diet could protect against cognitive decline through protection of the vascular system against damage [35], however a recent study of diet quality and cognitive function in 527 healthy adults reported that CVD risk factors did not significantly contribute to the observed relationship between a Mediterranean diet score index and cognition [36]. Oxidative stress and inflammation have also been implicated in Alzheimer’s disease [37] and can also be altered by dietary intake. The recommended food score, an index of dietary variety similar to that used in the current study was associated with intake of vitamin C, E, folate and antioxidants, and also lower plasma glucose, total serum cholesterol and blood pressure serum homocysteine and also lower C-reactive protein as markers of chronic disease and inflammation, in previous research [38]. Therefore there is likely to be multiple complex mechanisms underlying the association between diet quality and cognitive function.

This current study found participants who reported consumption of high fibre or multigrain bread options reported better cognitive function than participants who consumed white bread. A previous study of 178 institutionalised older people from Madrid found that higher consumption of carbohydrates and fibre was associated with better cognitive performance [39]. Higher fibre intake is also associated with other beneficial health effects in adults including reduced obesity, cardiovascular disease, diabetes, improving blood lipid profile and regulating blood glucose [40]. The higher fibre or multigrain bread options may be protecting against alterations in blood glucose metabolism and reducing diabetes risk, which have been implicated in cognitive decline and in the development of Alzheimer’s disease in the early stages [41].

Higher consumption of fluids in 2014 was also associated with better cognitive function in men in the current study. It should be noted that this item included only the drink types recommended in the Australian Dietary Guidelines including water, milk and soy milk, fruit and vegetable juice, low-joule cordial/soft drink, coffee and tea [25]. It did not include drinks classed as discretionary items due to their high sugar content including soft drinks, cordial, fruit drinks and flavoured milks or alcoholic beverages.

Many of the recommended drink types included in this item have been previously shown to be associated with cognitive function or dementia individually. In a recent meta-analysis of seven articles involving 10,941 participants, higher milk consumption was associated with reduced risk of cognitive disorders [42]. The role of coffee and tea consumption in the prevention of cognitive decline and dementia has also been investigated in systematic reviews, with mixed findings to date [43]. Higher consumption of fruit and vegetable juices was associated with reduced risk of Alzheimer’s disease in 1836 Japanese Americans after a 7–9 year follow-up in a longitudinal prospective cohort study [44]. It is likely that these individual items contributed to the associations observed between the general beverage item and cognitive function in this current study.

Additionally, greater consumption of water and other fluids may have displaced the consumption of discretionary items such as soft drink and alcoholic beverages from the diet. This may have had a positive effect on cognitive function, with animal studies linking soft drink consumption to memory deficits and the pathogenesis of Alzheimer’s disease [45], although human studies in this area have been limited and findings mixed to date [46, 47]. The relationship between alcohol intake and cognitive decline in the literature to date has been mixed, with some suggesting intake of alcohol to be protective only at low or moderate levels [48, 49], whilst heavy drinking at mid-life is thought to increase risk of cognitive impairment and dementia [50]. It should also be noted that although alcohol intake was investigated as a predictor of cognitive function in the current study, no significant associations were observed in any of the final models after adjustment for covariates.

Finally, in the current study, men who reported “usually” adding salt to their food during cooking displayed poorer cognitive function and reflects association observed between high sodium intake and poor cognitive function in a previous study [51]. A high sodium intake is linked to hypertension [52], which is itself an established risk factor for cognitive decline and dementia through cerebral vascular remodelling, reductions in cerebral perfusion and impairment of removal of potentially harmful proteins such as β-amyloid [35].

However, an unexpected finding was that women who reported “sometimes” adding salt to their food after cooking actually displayed better cognitive function than those who never added salt. Although surprising, a study by Rush et al. has previously reported an unexpected relationship between lower sodium intake assessed by FFQ and poorer cognitive performance in men and women over 50 years [53]. Although high levels of sodium intake are considered to be damaging to health, sodium is also an essential nutrient and low intakes in older adults have also been associated with cardiovascular disease and mortality [54], and Rush et al. proposed their a similar J-shaped association between sodium intake cognitive function [53]. However it should be noted that the current study assessed discretionary salt behaviour rather than total sodium intake or urinary sodium excretion [53]. However, in a previous Australian study, participants who reported adding salt to food at the table or during cooking also had higher urinary sodium excretion [55], direct comparison between these studies using differing methods of salt intake assessment is not possible. Due to the mixed findings observed between salt intake and cognitive function in the current study, further research using robust measures of sodium excretion are required.

The current study found inconsistent relationships between diet quality and cognitive function across the two time points investigated. This is despite diet quality scores remaining stable across the two time points, with little change in mean diet quality scores from 2010 to 2014. This is consistent with previous research which has shown diet quality remains stable over 1 to 8 years in adults [56, 57], although moderate changes have been observed previously after 10 years [58]. Studies should investigate tracking of diet quality including over longer time periods to assist in understanding how diet changes over time at this life stage and its relationship to health outcomes.

Differences in the relationships between diet quality, dietary items and cognitive function between men and women were observed in the current study. Although few studies of diet quality and cognitive function have split the analysis by gender, these findings add to a previous study which reported gender differences in this relationship in a cross-sectional study of 557 men and women aged 65 years and over residing in a small rural village in Greece [31]. Although the underlying causes of these observed differences are not clear, it is plausible that response biases in dietary reporting, and differences in trajectories of cognitive decline between men and women are factors [59]. Although TICS-m scores in the current study indicated a similar level of cognitive performance between men and women, DGI-2013 scores were approximately 5 points higher in women, indicating better diet quality in this group compared to men and consistent with this hypothesis. Therefore, further investigation of potential gender differences in the relationship between diet quality and cognitive function is warranted.

The strengths of our study include the detailed assessment of dietary intake and confounders at two time points, extensive participant information collected and the use of a validated diet quality index [6]. However, a number of limitations should be considered in the interpretation of this study’s findings. The sample size was relatively modest, which may have limited the detection of smaller associations reported by previous studies, although one of these was conducted in a sample of a similar size to the current study [31].The use of a non-quantified FFQ to assess dietary intake did not allow for exclusion of individuals with implausible energy intakes or adjustment of energy intake in the analysis. However, by adjusting for age, sex and physical activity key determinants of energy intake were considered in the regression models. Only a small proportion of the original cohort completed the cognitive assessment at follow up, which may have created a selection bias. Although the regression models were adjusted for potential confounders, it is possible some residual confounding remained from unmeasured variables such as self-efficacy, early-life education and experience and social connections. Finally, the relatively modest sample size may have limited our ability to detect the small associations reported by previous studies, although one of these was conducted in a sample of a similar size to the current study [31].

Cognitive function was assessed by a brief telephone-based method designed for use in epidemiological studies, however a longer face-to-face assessment with a battery of tests may have provided a more detailed and deeper assessment of cognitive function. Currently there are a multitude of cognitive assessment tests and batteries available and there is no recommended gold-standard test to use when investigating dietary intake and cognitive function relationships. Although many previous studies have used single measures of global cognitive function such as the MMSE, one study that did use a battery of 4 neuropsychological tests did find associations were not consistent across the tests [60], supporting this theory. Further research to determine the optimal battery of cognitive tests, in combination with emerging biomarkers and imaging techniques to determine cognitive impairment risk will help in this area [61].

These findings can be generalised to generally healthy urban and urban-fringe community-dwelling adults at the “peri-retirement” stage. The WELL study sample may have better health status than the general Australian population at this life stage, as indicated by reported higher reported scores on the RAND 36-item survey [9] compared to other Australian population-based samples [62, 63]. Also it should be noted that the subsample for this study was highly educated, with 45% having a university Bachelor degree or higher, compared to 27% in the original sample [12]. Being a sample of adults independently dwelling in the community, the findings of this sample are also not reflective of specific clinical populations with existing cognitive impairment.

Overall this study found limited and inconsistent evidence of an association between diet variety, dietary intake and cognitive function in mid and older aged adults. However it should be noted that adherence to a diet recommended by national dietary guidelines has been found to be associated with multiple positive health outcomes in adults previously including reduced obesity, hypertension [24, 64], chronic disease [8] and better quality of life [9], and is therefore likely to have health multiple benefits for mid and older aged adults other than the protection of cognitive function. Further research to investigate dietary patterns and cognitive function are recommended, and studies which investigate trajectories of dietary patterns and cognitive function over time periods of 10 years or more are crucial considering the long-term changes in cognitive function which are experienced by the growing ageing population worldwide.

## Conclusion

Evidence of an association between dietary variety and some limited dietary behaviours and cognitive function were observed, with variation by gender. Future research should consider trajectories of dietary change over longer time periods as determinants of health and function in older age.

## Supplementary information

**Additional file 1: Table S1.** Dietary Guideline Index (DGI-2013) components and scoring details. **Table S2.** Characteristics of included and excluded participants from the WELL Study, Victoria, Australia, 2010–2014. **Table S3.** Multivariate regression for DGI-2013 and components (2010) vs. TICS-m 2014 in the WELL study, participants with CVD excluded for sensitivity analysis. **Table S4.** Multivariate regression for DGI-2013 and components (2014) vs. TICS-m 2014 in the WELL study, participants with CVD excluded for sensitivity analysis.

## Data Availability

The datasets used and/or analysed during the current study are available from the corresponding author on reasonable request.

## Abbreviations

BMI: Body Mass Index
DGI-2013: Dietary Guideline Index
FFQ: Food Frequency Questionnaire
IPAQ-L: International Physical Activity Questionnaire
MET: Metabolic Equivalent of Task
MMSE: Mini-Mental State Examination
SEIFA: Socioeconomic Index for Areas Score
TICS-m: Telephone Interview for Cognitive Status
WELL: Wellbeing, Eating and Exercise for a Long Life Study
