# Supplementary material for: Diet quality and cognitive function in mid-aged and older men and women
Source: BMC Geriatr. 2019 Dec 21;19:361. doi: 10.1186/s12877-019-1326-5 (PMC6925482; doi:10.1186/s12877-019-1326-5)
Supplement: Supplementary file 1 — Additional file 1: Table S1. Dietary Guideline Index (DGI-2013) components and scoring details. Table S2. Characteristics of included and excluded participants from the WELL Study, Victoria, Australia, 2010–2014. Table S3. Multivariate regression for DGI-2013 and components (2010) vs. TICS-m 2014 in the WELL study, participants with CVD excluded for sensitivity analysis. Table S4. Multivariate regression for DGI-2013 and components (2014) vs. TICS-m 2014 in the WELL study, participants with CVD excluded for sensitivity analysis. [file 12877_2019_1326_MOESM1_ESM.docx]

Supplementary table 1. Dietary Guideline Index (DGI-2013) components and scoring details.

|  |  | DGI scoring details | | |  |
| --- | --- | --- | --- | --- | --- |
| Dietary component | Description | Criteria for max score | Criteria for min score | Max score | Item included in regression scores (potential score range). |
| 1. Food variety | Proportion of food from each of the 5 core food groups eater at least one serve per week. | 100% | 0% | 10 | Food variety continuous score (0-10). |
| 2. Vegetables | Servings per day. | Men ≥5.5, Women ≥5 | 0 | 10 | Servings per day (0-6). |
| 3. Fruit | Servings per day. | ≥2 | 0 | 10 | Servings per day (0-6). |
| 4. Grain (cereal) foods | Servings per day. | M ≥6, W ≥4 | 0 | 5 | Servings per day (0-7.92*^a^*). |
|  | Mostly wholegrain or high fibre cereals: Type of bread usually consumed. | Wholemeal/ rye bread | White bread/ none. | 5 | Type of bread consumed: White bread/none; High fibre white/multigrain/other; Wholemeal/ rye. |
| 5. Lean meat and poultry, fish, eggs, nuts and seeds, and legumes/beans | Servings per day. | M ≥2.5, W ≥2 | 0 | 5 | Servings per day (0-11.3*^a^*). |
|  | Proportion of lean meats and alternatives to total meat and alternatives per day. | 100% | 0% | 5 | N/A |
| 6. Milk, yoghurt, cheese and/or their alternatives | Servings per day. | M ≥2.5, W ≥4. | 0 | 10 | Servings per day (0-7.43*^a^*) |
| 7. Fluids | Total fluid intake (excluding soft drinks, cordial, fruit drinks, flavoured milks or alcoholic beverages) servings per day. | M ≥10, W ≥8 | 0 | 5 | Servings per day (0-18.6*^a^*) |
|  | Proportion of water to total fluid intake per day. | >50% | 0% | 5 | N/A |
| 8. Discretionary foods | Serves per day. | M ≤3; W ≤2.5 | M >3; W >2.5 | 10 | Serves per day (0-14.3*^a^*) |
| 9. Saturated fat | Trimming fat from meat. | Usually | Never or rarely | 5 | Trimming fat from meat: Never/rarely, Sometimes, Usually/always/I don’t eat meat |
|  | Type of milk usually consumed. | Skim or low fat milk | Whole milk | 5 | Type of milk consumed: Whole, Don’t know/soy, I don’t drink milk/low fat/skim |
| 10. Unsaturated spreads and oils | Servings per day. | M ≤4, W ≤2 | M >4, W>2 | 10 | Servings per day (0-7.86*^a^*) |
| 11. Added salt | Salt added to your food during cooking. | Never | Usually | 5 | Salt added during cooking: Never, Sometimes, Usually |
|  | Salt added to your food after it is cooked. | Never | Usually | 5 | Salt added after cooking: Never, Sometimes, Usually |
| 12. Added sugars | Servings per day. | M ≤1.5, F ≤1.25 | M >1.5, F >1.25 | 10 | Servings per day (0-4.07*^a^*) |
| 13. Alcohol | Servings per day. | ≤2 | >2 | 10 | Servings per day (0-6.13*^a^*) |

*^a^Highest reported score in sample at baseline 2010.*

*M, Men; W, Women; N/A, not included as an individual exposure in linear regressions as item already considered in another component.*

Supplementary table 2. Characteristics of included and excluded participants from the WELL Study, Victoria, Australia, 2010-2014.

|  | Included n = 617 | Excluded n = (101-135)^a^ |
| --- | --- | --- |
|  | Mean (SD) | Mean (SD) |
| Age T1 (years) | 60.2 (3.14) | 60.5 (3.01) |
| BMI T1 (kg/m^2^) | 26.6 (4.71) | 26.2 (4.46) |
| BMI T3 (kg/m^2^) | 26.5 (4.76) | 26.7 (5.64) |
| Total physical activity T1  (MET hours/week) | 93.1 (78.9) | 85.3 (71.4) |
| Total physical activity T3  (MET hours/week) | 93.8 (82.4) | 107.6 (105.6) |
| Dietary Guideline Index T1 | 87.5 (13.9) | 85.0 (15.7) |
| Dietary Guideline Index T3 | 88.2 (13.3) | 85.0 (13.4)* |
| Geriatric depression scale T3 | 1.72 (2.30) | 1.86 (2.47) |
| TICS-M T3 | 37.0 (4.06)  Min – 24  Max - 48 | 35.0 (5.46)**  Min – 17  Max - 47 |
| Region T1 | N (%) | N (%) |
| *Urban* | 598 (96.9) | 127 (94.1) |
| *Rural* | 19 (3) | 8 (5.93) |
| Country of birth |  |  |
| *Australia* | 472 (76.5) | 100 (75.2) |
| *UK* | 49 (7.94) | 9 (6.77) |
| *Other* | 96 (15.5) | 24 (18.1) |
| Relationship status T1 |  |  |
| *Married/defacto* | 477 (77.4) | 100 (74.1) |
| *Separated/divorced* | 82 (13.3) | 18 (13.3) |
| *Widowed* | 28 (4.55) | 10 (7.41) |
| *Never married* | 29 (4.71) | 7 (5.19) |
| Retired T1 |  |  |
| *Yes* | 215 (35.3) | 45 (35.2) |
| *No* | 395 (64.8) | 83 (64.8) |
| Education |  |  |
| *Up to 10 years* | 136 (22.0) | 42 (32.1)* |
| *12 years/trade/certificate* | 204 (33.1) | 39 (29.8) |
| *University degree* | 277 (44.9) | 50 (38.2) |
| Smoking status T1 |  |  |
| *Never smoked* | 346 (56.1) | 61 (45.9) |
| *Former smoker* | 207 (33.6) | 55 (41.4) |
| *Daily smoker* | 64 (10.4) | 17 (12.8) |
| History of stroke, n (%) | 18 (2.9) | 5 (3.70) |
| History of diabetes, n (%) | 52 (8.4) | 13 (9.63) |
| History of heart disease, n (%) | 112 (18.2) | 22 (16.3) |
| History of hypertension, n (%) | 276 (44.7) | 65 (48.2) |

^a^ n ranges from 111- to 111 due to missing responses.

*P<0.05, **P<0.01 compared to included group.

Supplementary Table 3. Multivariate regression for DGI-2013 and components (2010) vs. TICS-m 2014 in the WELL study, participants with CVD excluded for sensitivity analysis

|  | **Total (n=492)** | **Men (n=222)** | **Women (n=270)** |
| --- | --- | --- | --- |
|  | **B (95% CI)** | **B (95% CI)** | **B (95% CI)** |
| **DGI-2013** |  |  |  |
| crude | 0.02 (-0.00, 0.05) | 0.01 (-0.03, 0.05) | 0.01 (-0.03, 0.05) |
| Adjusted | 0.00 (-0.03, 0.03) | 0.01 (-0.03, 0.05) | -0.00 (-0.04, 0.04) |
| **Dietary variety** |  |  |  |
| crude | 0.35 (0.05, 0.65)* | 0.30 (-0.19, 0.79) | 0.25 (-0.13, 0.62) |
| Adjusted | 0.22 (-0.06, 0.50) | 0.21 (-0.21, 0.64) | 0.22 (-0.15, 0.58) |
| **Vegetable serves** |  |  |  |
| crude | 0.23 (-0.08, 0.53) | 0.04 (-0.35, 0.44) | 0.10 (-0.40, 0.60) |
| Adjusted | 0.14 (-0.18, 0.45) | 0.13 (-0.26, 0.53) | 0.18 (-0.28, 0.64) |
| **Fruit serves** |  |  |  |
| crude | -0.02 (-0.26, 0.22) | -0.10 (-0.56, 0.36) | -0.19 (-0.53, 0.16) |
| Adjusted | -0.13 (-0.39, 0.13) | -0.07 (-0.48, 0.35) | -0.18 (-0.51, 0.15) |
| **Grain foods serves** |  |  |  |
| crude | -0.10 (-0.48, 0.29) | 0.10 (-0.21, 0.41) | -0.08 (-0.63, 0.47) |
| Adjusted | -0.02 (-0.36, 0.31) | 0.08 (-0.25, 0.40) | -0.19 (-0.65, 0.28) |
| **Wholegrain bread** |  |  |  |
| Crude |  |  |  |
| *“I don’t eat bread/white bread”* | ref | ref | ref |
| *“high fibre white/multigrain/other”* | 1.50 (0.43, 2.57)** | 0.67 (-0.69, 2.03) | 2.09 (0.24, 3.94)* |
| *“Wholemeal/rye”* | -0.37 (-0.89, 1.63) | -0.79 (-2.47, 0.88) | 1.50 (-0.19, 3.20) |
| Adjusted |  |  |  |
| *“I don’t eat bread/white bread”* | ref | ref | ^ref^ |
| *“high fibre white/multigrain/other”* | 0.98 (-0.10, 2.05) | 0.52 (-0.78, 1.81) | 1.61 (0.06, 3.16)* |
| *“Wholemeal/rye”* | -0.06 (-1.32, 1.20) | -0.87 (-2.55, 0.81) | 0.88 (-0.62, 2.38) |
| **Meat and alternatives serves** |  |  |  |
| Crude | 0.16 (-0.19, 0.50) | 0.21 (-0.23, 0.65) | 0.03 (-0.34, 0.41) |
| Adjusted | 0.04 (-0.31, 0.39) | 0.16 (-0.24, 0.57) | -0.01 (-0.40, 0.37) |
| **Dairy serves** |  |  |  |
| crude | 0.22 (0.01, 0.44)* | 0.14 (-0.29, 0.58) | -0.24 (-0.16, 0.64) |
| Adjusted | 0.17 (-0.03, 0.38) | 0.02 (-0.38, 0.43) | 0.27 (-0.09, 0.62) |
| **Fluid serves** |  |  |  |
| crude | 0.06 (-0.03, 0.16) | 0.08 (-0.07, 0.22) | 0.01 (-0.12, 0.14) |
| Adjusted | -0.01 (-0.11, 0.08) | 0.01 (-0.15, 0.17) | -0.05 (-0.17, 0.08) |
| **Discretionary foods serves** |  |  |  |
| crude | -0.08 (-0.23, 0.08) | 0.08 (-0.10, 0.26) | -0.07 (-0.25, 0.11) |
| Adjusted | 0.02 (-0.13, 0.16) | 0.07 (-0.11, 0.25) | -0.04 (-0.21, 0.14) |
| **Trimming fat from meat** |  |  |  |
| Crude |  |  |  |
| *“Never/rarely”* | ref | ref | ref |
| *“Sometimes”* | -0.18 (-2.31, 1.94) | -0.72 (-3.42, 1.97) | 0.60 (-3.12, 4.31) |
| *“Usually/always/I don’t eat meat”* | 0.83 (-1.03, 2.68) | 0.03 (-2.31, 2.37) | 1.31 (-1.80, 4.41) |
| Adjusted |  |  |  |
| *“Never/rarely”* | ref | Ref | Ref |
| *“Sometimes”* | -0.75 (-2.55, 1.05) | -1.50 (-3.96, 0.97) | 0.14 (-3.38, 3.66) |
| *“Usually/always/I don’t eat meat”* | -0.17 (-1.74, 1.39) | -0.63 (-2.75, 1.50) | 0.58 (-2.27, 3.43) |
| **Type of milk drunk** |  |  |  |
| Crude |  |  |  |
| *“Whole”* | ref | ref | ref |
| *“Don’t know/soy”* | 0.20 (-1.49, 1.89) | -1.68 (-4.27, 0.91) | 0.34 (-1.94, 2.63) |
| *“I don’t drink milk/low fat/skim”* | 0.03 (-0.86, 0.91) | -0.51 (-1.56, 0.53) | 0.10 (-1.55, 1.76) |
| Adjusted |  |  |  |
| *“Whole”* | ref | Ref | Ref |
| *“Don’t know/soy”* | -0.71 (-2.06, 0.64) | -1.68 (-4.61, 1.26) | -0.32 (-2.32, 1.68) |
| *“I don’t drink milk/low fat/skim”* | -0.45 (-1.37, 0.47) | -0.44 (-1.41, 0.54) | -0.46 (-2.12, 1.20) |
| **Unsaturated fat oils and spreads serves** |  |  |  |
| crude | 0.39 (0.02, 0.77) | 0.45 (0.03, 0.87)* | 0.34 (-0.11, 0.78) |
| Adjusted | 0.27 (-0.12, 0.66) | 0.33 (-0.05, 0.71) | 0.24 (-0.23, 0.72) |
| **Salt added after cooking** |  |  |  |
| Crude |  |  |  |
| *“Never”* | ref | ref | ref |
| *“Sometimes”* | 0.34 (-0.48, 1.15) | -0.42 (-1.48, 0.63) | 0.96 (-0.01, 1.92) |
| *“Usually”* | 0.31 (-0.51, 1.13) | 0.14 (-1.29, 1.57) | 0.62 (-0.26, 1.50) |
| Adjusted |  |  |  |
| *“Never”* | ref | ref | Ref |
| *“Sometimes”* | 0.51 (-0.15, 1.17) | -0.14 (-1.20, 0.92) | 1.04 (0.17, 1.91)* |
| *“Usually”* | 0.66 (-0.12, 1.45) | 0.66 (-0.73, 2.05) | 0.66 (-0.23, 1.55) |
| **Salt added during cooking** |  |  |  |
| Crude |  |  |  |
| *“Never”* | ref | ref | Ref |
| *“Sometimes”* | 0.22 (-0.47, 0.91) | 1.21 (0.02, 2.03)* | -0.42 (-1.65, 0.80) |
| *“Usually”* | -0.79 (-1.96, 0.39) | -0.97 (-2.60, 0.65) | -0.52 (-2.09, 1.05) |
| *“Don’t know”* | -1.38 (-3.85, 1.09) | -0.29 (-2.66, 2.08) | n/a |
| Adjusted |  |  |  |
| *“Never”* | ref | ref | Ref |
| *“Sometimes”* | 0.29 (-0.34, 0.93) | 0.96 (-0.11, 2.03) | -0.37 (-1.60, 0.85) |
| *“Usually”* | -0.73 (-1.76, 0.30) | -1.09 (-2.64, 0.46) | -0.40 (-1.79, 1.00) |
| *“Don’t know”* | -0.33 (-2.43, 1.77) | -0.08 (-2.37, 2.20) | n/a |
| **High sugar food serves** |  |  |  |
| crude | -0.43 (-0.83, -0.03)* | -0.25 (-0.75, 0.25) | -0.37 (-1.09, 0.34) |
| Adjusted | -0.20 (-0.52, 0.12) | -0.19 (-0.62, 0.24) | -0.20 (-0.80, 0.40) |
| **Alcohol serves** |  |  |  |
| crude | 0.10 (-0.16, 0.36) | 0.26 (0.02, 0.50)* | 0.32 (-0.30, 0.94) |
| Adjusted | 0.25 (-0.00, 0.50) | 0.30 (0.05, 0.55)* | 0.23 (-0.30, 0.77) |

*Adjusted for age (sex – total only) + education +urban/rural + clustering by postcode + total physical activity (T1)*

*DGI-2013, Dietary Guideline Index; TICS-m, Telephone Interview of Cognitive Status.*

*^a^Results presented with outliers removed, *P<0.05, **P<0.01.*

Supplementary Table 4. Multivariate regression for DGI-2013 and components (2014) vs. TICS-m 2014 in the WELL study, participants with CVD excluded for sensitivity analysis

|  | Total (n=617) | Men (n=302) | Women (n=315) |
| --- | --- | --- | --- |
|  | B (95% CI) | B (95% CI) | B (95% CI) |
| **DGI-2013** |  |  |  |
| crude | 0.03 (-0.01, 0.06) | 0.04 (-0.00, 0.07) | -0.01 (-0.07, 0.04) |
| Adjusted | 0.01 (-0.03, 0.04) | 0.03 (-0.01, 0.07) | -0.02 (-0.06, 0.03) |
| **Dietary variety** |  |  |  |
| crude | 0.04 (-0.28, 0.37) | 0.15 (-0.27, 0.57) | -0.19 (-0.61, 0.23) |
| Adjusted | 0.00 (-0.29, 0.29) | 0.16 (-0.18, 0.50) | -0.18 (-0.57, 0.21) |
| **Vegetable serves** |  |  |  |
| crude | 0.32 (0.06, 0.59)* | 0.17 (-0.24, 0.58) | 0.21 (-0.13, 0.56) |
| Adjusted | 0.17 (-0.11, 0.45) | 0.17 (-0.20, 0.55) | 0.20 (-0.16, 0.57) |
| **Fruit serves** |  |  |  |
| crude | -0.05 (-0.32, 0.23) | -0.02 (-0.40, 0.35) | -0.30 (-0.94, 0.34) |
| Adjusted | -0.14 (-0.43, 0.16) | -0.03 (-0.33, 0.38) | -0.32 (-0.92, 0.28) |
| **Grain foods serves** |  |  |  |
| crude | -0.28 (-0.29, 0.03) | 0.24 (-0.12, 0.59) | -0.62 (-1.09, -0.14)* |
| Adjusted | -0.17 (-0.48, 0.14) | 0.12 (-0.23, 0.48) | -0.52 (-1.03, -0.01)* |
| **Wholegrain bread** |  |  |  |
| Crude |  |  |  |
| *“I don’t eat bread/white bread”* | ref | ref | ref |
| *“high fibre white/multigrain/other”* | 1.46 (0.41, 2.52)* | 1.63 (0.32, 2.94)* | 1.20 (-0.49, 2.89) |
| *“Wholemeal/rye”* | 1.21 (0.14, 2.28)* | 0.55 (-1.21, 2.32) | 1.75 (0.11, 3.40)* |
| Adjusted |  |  |  |
| *“I don’t eat bread/white bread”* | ref | ref | Ref |
| *“high fibre white/multigrain/other”* | 0.89 (-0.15, 1.93) | 1.22 (-0.10, 2.54) | 0.74 (-0.88, 2.36) |
| *“Wholemeal/rye”* | 0.37 (-0.70, 1.44) | 0.04 (-1.59, 1.67) | 0.72 (-0.70, 2.15) |
| **Meat and alternatives serves** |  |  |  |
| crude | 0.13 (-0.22, 0.49) | 0.07 (-0.44, 0.58) | 0.10 (-0.35, 0.55) |
| Adjusted | 0.10 (-0.22, 0.41) | 0.09 (-0.38, 0.55) | 0.11 (-0.26, 0.49) |
| **Dairy serves** |  |  |  |
| crude | 0.15 (-0.01, 0.32) | 0.31 (0.06, 0.56)* | -0.00 (-0.36, 0.35) |
| Adjusted | 0.15 (-0.03, 0.34) | 0.20 (-0.04, 0.45) | 0.12 (-0.26, 0.49) |
| **Fluid serves** |  |  |  |
| crude | 0.08 (-0.04, 0.20) | 0.21 (0.09, 0.33)** | -0.06 (-0.24, 0.11) |
| Adjusted | 0.04 (-0.07, 0.16) | 0.15 (0.02, 0.28)* | -0.03 (-0.21, 0.15) |
| **Discretionary foods serves** |  |  |  |
| crude | -0.08 (-0.29, 0.12) | -0.02 (-0.21, 0.18) | 0.05 (-0.29, 0.40) |
| Adjusted | 0.01 (-0.20, 0.23) | -0.01 (-0.21, 0.19) | 0.04 (-0.31, 0.39) |
| **Trimming fat from meat** |  |  |  |
| Crude |  |  |  |
| *“Never/rarely”* | ref | ref | ref |
| *“Sometimes”* | 0.64 (-0.95, 2.24) | 0.50 (-1.39, 2.38) | 0.83 (-1.85, 3.51) |
| *“Usually/always/I don’t eat meat”* | 0.60 (-0.53, 1.73) | -0.29 (-1.69, 1.11) | 1.20 (-0.31, 2.72) |
| Adjusted |  |  |  |
| *“Never/rarely”* | ref | Ref | ref |
| *“Sometimes”* | 1.08 (-0.49, 2.65) | 1.24 (-0.62, 3.11) | 0.78 (-1.77, 3.34) |
| *“Usually/always/I don’t eat meat”* | 0.23 (-0.67, 1.12) | -0.15 (-1.41, 1.11) | 0.68 (-0.87, 2.24) |
| **Type of milk drunk** |  |  |  |
| Crude |  |  |  |
| *“Whole”* | ref | ref | ref |
| *“Don’t know/soy”* | -0.26 (-1.49, 0.97) | -1.67 (-3.62, 0.28) | -1.48 (-3.48, 0.53) |
| *“I don’t drink milk/low fat/skim”* | 0.34 (-0.36, 1.05) | 0.43 (-0.59, 1.44) | 0.25 (-0.69, 1.18) |
| Adjusted |  |  |  |
| *“Whole”* | ref | ref | ref |
| *“Don’t know/soy”* | -0.70 (-2.04, 0.64) | -1.48 (-3.48, 0.53) | -0.57 (-2.48, 1.34) |
| *“I don’t drink milk/low fat/skim”* | 0.02 (-0.71, 0.75) | 0.25 (-0.69, 1.18) | -0.19 (-1.42, 1.04) |
| **Unsaturated fat oils and spreads serves** |  |  |  |
| crude | 0.23 (-0.15, 0.60) | -0.06 (-0.53, 0.42) | 0.69 (0.22, 1.17)** |
| Adjusted | 0.14 (-0.23, 0.52) | -0.13 (-0.57. 0.32) | 0.53 (0.04, 1.02)* |
| **Salt added after cooking** |  |  |  |
| Crude |  |  |  |
| *“Never”* | ref | ref | ref |
| *“Sometimes”* | 0.14 (-0.70, 0.99) | -0.39 (-1.081, 1.02) | 0.54 (-0.49, 1.57) |
| *“Usually”* | 0.05 (-0.88, 0.98) | 0.00 (-1.50, 1.51) | 0.38 (-0.62, 1.38) |
| Adjusted |  |  |  |
| *“Never”* | Ref | Ref | Ref |
| *“Sometimes”* | 0.25 (-0.55, 1.06) | -0.15 (-1.51, 1.21) | 0.55 (-0.43, 1.53) |
| *“Usually”* | 0.51 (-0.40, 1.42) | 0.39 (-1.11, 1.90) | 0.60 (-0.31, 1.51) |
| **Salt added during cooking** |  |  |  |
| Crude |  |  |  |
| *“Never”* | ref | ref | ref |
| *“Sometimes”* | 0.02 (-0.98, 1.03) | -0.08 (-1.40, 1.24) | 0.20 (-0.99, 1.39) |
| *“Usually”* | -1.27 (-2.39, -0.14)* | -1.57 (-2.80, -0.34)* | -0.92 (-2.48, 0.63) |
| *“Don’t know”* | -1.33 (-6.35, 3.70) | -0.63 (-5.76, 4.49) | n/a |
| Adjusted |  |  |  |
| *“Never”* | ref | Ref | Ref |
| *“Sometimes”* | -0.15 (-1.00, 0.71) | -0.61 (-1.72, 0.50) | 0.28 (-0.87, 1.43) |
| *“Usually”* | -1.08 (-1.96, -0.20)* | -1.59 (-2.74, -0.45)** | -0.56 (-1.85, 0.72) |
| *“Don’t know”* | 0.03 (-4.70, 4.76) | -0.24 (-4.97, 4.48) | n/a |
| **High sugar food serves** |  |  |  |
| crude | -0.45 (-1.03, 0.14) | -0.26 (-0.88, 0.36) | -0.26 (-1.63, 1.11) |
| Adjusted | -0.23 (-0.81, 0.35) | -0.15 (-0.72, 0.42) | -0.36 (-1.55, 0.83) |
| **Alcohol serves** |  |  |  |
| crude | 0.03 (-0.27, 0.33) | 0.09 (-0.26, 0.44) | 0.48 (-0.00, 0.95) |
| Adjusted | 0.30 (-0.06, 0.65) | 0.16 (-0.24, 0.56) | 0.61 (-0.00, 1.23) |

*Adjusted for age (sex – total only) + education +urban/rural + clustering by postcode + total physical activity (T1).*

*DGI-2013, Dietary Guideline Index; TICS-m, Telephone Interview of Cognitive Status.*

*^a^Results presented with outliers removed, *P<0.05, **P<0.01.*
